# Parents’ Decisions to Vaccinate Children against COVID-19: A Scoping Review

**DOI:** 10.3390/vaccines9121476

**Published:** 2021-12-14

**Authors:** Fengming Pan, Hongyu Zhao, Stephen Nicholas, Elizabeth Maitland, Rugang Liu, Qingzhen Hou

**Affiliations:** 1 Department of Biostatistics, School of Public Health, Cheeloo College of Medicine, Shandong University, Jinan 250012, China; pfengming@mail.sdu.edu.cn (F.P.); zhaohongyuu2019@mail.sdu.edu.cn (H.Z.); 2 National Institute of Health Data Science of China, Shandong University, Jinan 250002, China; 3 Australian National Institute of Management and Commerce, Sydney, NSW 2015, Australia; stephen.nicholas@newcastle.edu.au; 4 Research Institute for International Strategies, Guangdong University of Foreign Studies, Guangzhou 510420, China; 5 School of Economics and School of Management, Tianjin Normal University, Tianjin 300074, China; 6 Newcastle Business School, University of Newcastle, Newcastle, NSW 2308, Australia; 7 School of Management, University of Liverpool, Chatham Building, Chatham Street, Liverpool L69 7ZH, UK; e.maitland@liverpool.ac.uk; 8 School of Health Policy & Management, Nanjing Medical University, Nanjing 211166, China; 9 Center for Global Health, Nanjing Medical University, Nanjing 211166, China; 10 Institute of Healthy Jiangsu Development, Nanjing Medical University, Nanjing 211166, China

**Keywords:** COVID-19, vaccine hesitancy, acceptance, willingness, children, scoping review

## Abstract

Since 2019, the COVID-19 pandemic has resulted in sickness, hospitalizations, and deaths of the old and young and impacted global social and economy activities. Vaccination is one of the most important and efficient ways to protect against the COVID-19 virus. In a review of the literature on parents’ decisions to vaccinate their children, we found that widespread vaccination was hampered by vaccine hesitancy, especially for children who play an important role in the coronavirus transmission in both family and school. To analyze parent vaccination decision-making for children, our review of the literature on parent attitudes to vaccinating children, identified the objective and subjective influencing factors in their vaccination decision. We found that the median rate of parents vaccinating their children against COVID-19 was 59.3% (IQR 48.60~73.90%). The factors influencing parents’ attitudes towards child vaccination were heterogeneous, reflecting country-specific factors, but also displaying some similar trends across countries, such as the education level of parents. The leading reason in the child vaccination decision was to protect children, family and others; and the fear of side effects and safety was the most important reason in not vaccinating children. Our study informs government and health officials about appropriate vaccination policies and measures to improve the vaccination rate of children and makes specific recommendations on enhancing child vaccinate rates.

## 1. Introduction

In September 2021, the World Health Organization (WHO) [1] reported 230 million COVID-19 cases and 4.7 million COVID-19 deaths globally. In the USA, children made up 15.5% of all infected people by September 2021 and after declining in the early summer of 2021, child COVID-19 infections increased exponentially, accounting for 28.9% of all weekly reported new COVID-19 cases. Considering that children make up 22.2 percent of the US population, there are more newly infected children than people of other ages [2]. One side effect of Covid-19 has been pressure on routine healthcare, especially on childhood vaccine and influenza vaccine programs [3,4,5] and pediatric clinics [6,7]. In order to prevent the spread of the virus, many countries have adopted home quarantine and social distancing policies [8], which have seriously affected children’s outdoor activities and normal schooling [9]. Although quarantine strategies reduced the infectious risk in the short run [10,11], the pandemic staged comebacks when the bans were lifted and may have long-term effects on children’s mental health and well-being [12].

The morbidity and mortality of children infected with COVID-19 is lower than that of adults, and the clinical symptoms are milder [13,14,15,16,17,18,19], but children are still at risk of COVID-19 infection and may act as virus transmitters at home and at school [20,21]. In addition, the continuing mutation of coronavirus strains might increase COVID-19′s infection rate and virulence in children [22], challenging existing prevention and control measures. The vaccination of juveniles is an important part of developing herd immunity, allowing the opening up of societies [23]. For these reasons, vaccinating children against COVID-19 [24] is an essential element to combatting the epidemic in the long term [25,26].

To eliminate the COVID-19 pandemic, there are more than 300 COVID-19 vaccines developed worldwide, 121 of which are in clinical trials [27]. Although the virus has evolved into multiple variants, with the Delta variant currently dominant in most regions of the world [28], many studies have shown that the existing COVID-19 vaccines largely preserved neutralizing titers with slightly lower or unchanged efficacy [22], showing substantial protection. Moreover, the results of clinical trials have shown that several vaccines are safe and effective in preventing COVID-19 in children, such as CoronaVac, Pfizer/BioNTech (BNT162b2), and Moderna (mRNA-1273) [29,30,31]. Approval has been granted for vaccination of children 5 through 17 years old in many countries. China has approved the two COVID-19 inactivated vaccines from Sinopharm and Sinovac for emergency use in children aged 3–17 years [32] and the FDA has approved the Pfizer/BioNTech for emergency use in children 5–17 year old [33,34].

However, the promotion of COVID-19 vaccines is still threatened by vaccine hesitancy, which is a large obstacle for the success of vaccine [35]. Whether children are vaccinated mainly depends on their parents; therefore, it is important to understand parents’ decisions to vaccinate their children against COVID-19. To assess parent willingness to vaccinate their children, we reviewed recent studies about the attitudes of parents towards the COVID-19 vaccines for their children and the factors influencing the vaccination decision. This scoping review aims to help governments develop policies to improve the public acceptance of vaccines for children. 

## 2. Materials and Methods

The literature review was conducted according to the Preferred Reporting Items for Systematic Reviews and Meta-Analyses extension for Scoping Reviews (PRISMA-ScR) Statement [36] (see Appendix A). The study has no written or published priori protocols. Our research questions were:

1. What were parents’ attitudes towards having their children vaccinated against COVID-19?
2. What factors and reasons influenced parents’ willingness to make the vaccination decision?

### 2.1. Search Strategy and Data Source

The PubMed, Embase, Web of Science and Cochrane Central electronic databases were searched in July 2021 to find all potentially relevant articles without restrictions. The search strategy is described in Table 1. In brief, the keywords used were: COVID-19 and its’ synonyms, vaccin*, immunization, child* or parents, hesitancy and its’ synonyms or antonyms, published from 2019 to 2021.

### 2.2. Inclusion and Exclusion Criteria

The inclusion criteria were based on participant, outcome, and study design. The eligibility criteria of the participant was defined as adults older than 16 years and the outcome must include the attitude to COVID-19 vaccines for children. The origin studies were limited to English language articles published between December 2019 and 25 July 2021. We excluded: duplicate records, non-original research, studies with unrelated topics, and undefined outcomes.

### 2.3. Data Extraction and Quality Assessment

The initial literature scanning was independently conducted by two authors, based on the article titles and abstracts. All the studies meeting the requirements were exported to Excel, ensuring the removal of any duplicates. Next, each author was given a specific number of articles to read the full text to verify the inclusion decision. In this process, we designed a chart to organize all details including the first author of the study, publication year, sample size, the type of participants, the age of participants, the sex of participants, study design, sampling method, study setting, country of participants, the rate of parents’ willingness to have their children vaccinated against COVID-19, the factors influencing the rate and the reasons for the vaccination decision. We combined the factors and reasons with similar meanings. During the study selection and evaluation process, the first author was responsible for resolving any differences and final evaluation.

### 2.4. Data Analysis

The factors that influence parents’ attitudes towards COVID-19 vaccines for children were categorized and only those with *p* < 0.05 were included. Meta-analysis was not performed due to heterogeneity in the types of subjects in our review.

## 3. Results

**Figure 1:**
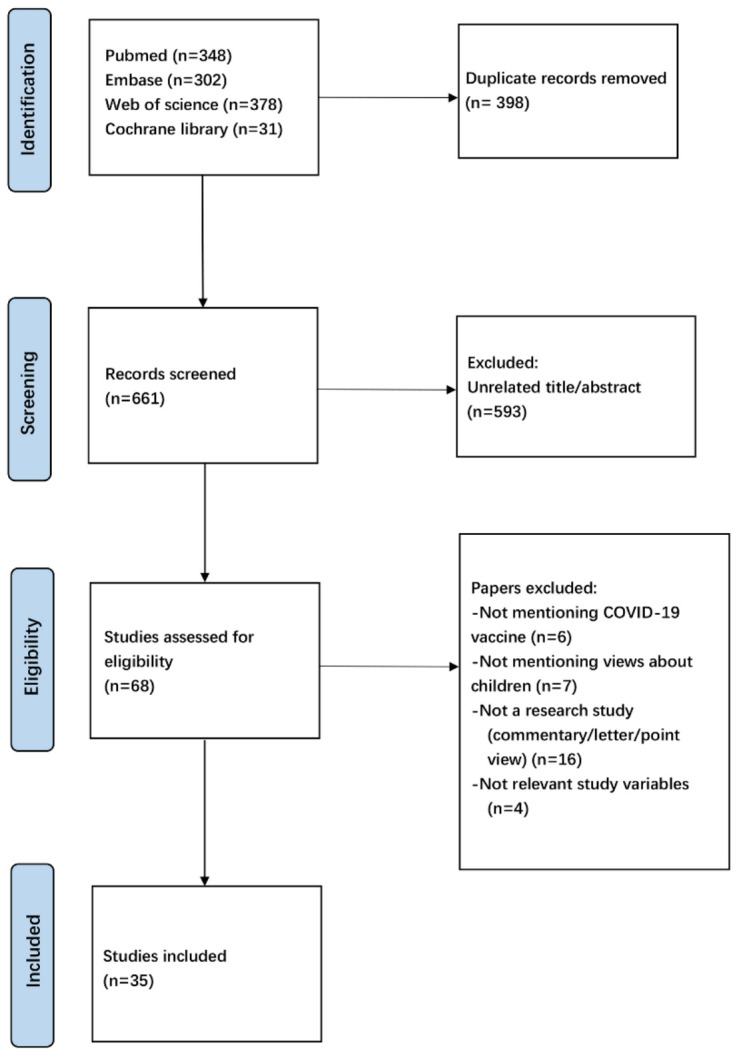
PRISMA flow diagram of study selection.

In total, 1059 records were retrieved from the electronic database search (348 in PubMed, 302 in Embase, 378 in Web of science, 31 in Cochrane Library). There were 661 records left after removing the duplicates. After checking the titles and abstracts, 593 were excluded; the remaining 68 full-text articles were assessed for eligibility, and 35 studies were reserved for this review. In Figure 1, the PRISMA diagram describes the study selection and exclusion process.

### 3.1. Study Characteristics

Table 2 displays the 34 cross-sectional [35,37,38,39,40,41,42,43,44,45,46,47,48,49,50,51,52,53,54,55,56,57,58,59,60,61,62,63,64,65,66,67,68,69] and one experimental study [70]. Twenty-eight papers were published in 2021 and seven in 2020, with the majority focused on parents and two focused on general adults to estimate their attitudes towards children’s vaccination against COVID-19 [42,58]. Sample size involved in studies range from 25 participants [61] to 17871 participants [44]. The participants were mainly 30–40 years old females, with two studies not clearly describing the age of the participants [35,42]. Most of the studies were conducted in one country, one study covered six countries [39], and two studies were global surveys [44,60]. Online surveys conducted through Facebook, Qualtrics, Google and similar platforms were the most common methods for data collection, resulting in non-probability samples. Stratified sampling was adopted by two studies [48,52], the snowball method in another two [60,61], and the remaining used random sampling. Offline data collection methods [39,47,48] and phone interviews [41,58,61] were undertaken by three studies each. Four studies contacted participants by email [43,49,50,56].

### 3.2. Parents Decision to Vaccinate the Children against COVID-19

Twenty-five studies reported a 10.4% to 92% willingness rate of parents to vaccinate the children against COVID-19, with an additional study not clearly reporting the willingness rate [65]. Among the 25 studies, rates were highly heterogeneous, and the median (unadjusted) rate was 59.3% (IQR 48.6~73.9%). As a reference, the median rate of parents’ willingness to vaccinate themselves was 61.4% (IQR 50.3~78.9%). There are also eight results with limited conditions, and one qualitative interview study. 

Overall, we found:

Seven USA studies reported overall rates and three reported conditional rates. Seven USA studies reported the rates to vaccinate children ranging from 21.6% to 70.1% [42,43,46,50,63,64,66], four of which had a sample size of more than 1000 with the acceptance rate over 50%. A Gallup Panel web study reported a willingness to vaccinate acceptance of 48.6% [42]. Two papers, parents of patients at Cincinnati Children’s Hospital Medical Center [46] and parents of Amish families [43], with low sample sizes and smaller populations (<500) reported low willingness to vaccinate rates of 21.6% and 24.3% respectively. In particular, a study of mothers with a mental health history [53] concluded that mothers with a post-traumatic stress disorder (PTSD) history were more reluctant to vaccinate than mothers without a PTSD history. One paper [70] found that regardless of the treatment children given, 19.7% of those parents did not plan to vaccinate their children. Kimberly et al. [61] qualitatively explored maternal willingness to accept a COVID-19 vaccine, finding a 16% willingness to vaccinate rate.

Four Chinese studies [48,57,58,59] reported overall rates and one reported a conditional rate, with a willingness to vaccinate rate ranging from 44.5% to 85.3%. The lowest 44.5% willingness to vaccinate rate involved healthcare workers of five collaborative hospitals located in three Chinese provinces and the highest score, 85.3%, were among guardians who visited community health centers in the Xuhui District, Shanghai. Another Shanghai study [52] investigated acceptance at different effectiveness and safety profiles, with acceptance rates ranging from 31.3% to 87.5%.

Four Turkish studies reported overall rates and two reported conditional rates. Adopting non-probability sampling methods, four Turkish studies [37,51,54,68] reported the acceptance rates for children of 10.4%, 36.3%, 73.9% and 75%, with the highest rate focusing on pediatrics. In two studies with conditional vaccination willingness rates, Büşra et al. [41] found that the acceptance rate rose from 38.4% to 41.9% when the vaccine was free. Some parents preferred national vaccines (56.8%) over foreign vaccines (28.9%) [47].

Three Italian studies reported overall rates. Bologna residents showed a willingness to vaccinate rate of 60.4% [49], a study conducted in 20 regions in Italy showed a willingness to vaccinate rate of 91.1% [38], and the willingness to vaccinate of participants from Naples was particularly low—17.2% [69].

The following countries appeared only once in the collected studies: a Germany [55] study showed an acceptance rate of 51% for parents to vaccinate their children; a study in Calgary, Canada, in which all participants were women, had a willingness rate of 60.4% [56]; the willingness to vaccinate among physicians in Colombia was 85.7% [45]; and an online cross-sectional survey in England reported a willingness to vaccinate rate of 89.1% [40]. The acceptance reached 92%, when parents whose children had already received a campaign dose of MR vaccine at vaccination sites in Zambia [62]. Israel’s researchers divided their subjects by occupation and found that the acceptance rate to vaccinate children was 70% for general population, 60% for doctors and 55% for nurses [35] and 46% participants from Qatar University stated they would not vaccinate their children [67].

Some studies were conducted in more than one country. A study conducted of caregivers in pediatric Emergency Departments (ED) across six countries had a willingness to vaccinate rate of 65.2% [39]. The snowball method was used for the study of Ruggiero et al. [60], which showed a population willingness to vaccinate rate of 49.5%, but did not mention the specific country of origin. Assuming the effectiveness of vaccines, a study conducted in 16 countries [44] among women aged 18 years or older, currently pregnant or with at least one child under 18 years of age, had a 69.2% willing to vaccine their children when efficacy was 90%.

### 3.3. Factors Shaping Parental Decisions to Vaccinate against COVID-19

Twelve studies [35,37,45,46,51,52,61,66,67,68,69,70] did not mention the factors or undertake statistical tests on the factors influencing parental decisions toward COVID-19 vaccines for their children, 16 papers used univariable analysis to assess the factors influencing parental decisions towards COVID-19 vaccinations, and 17 papers undertook multivariable analysis. Table 3 and Table 4 reports the influencing factors and divides them into objective and subjective factors.

Objective factors in Table 3 were mostly related to individual characteristics, with education being the most common factor—tested in nine univariable studies [41,42,44,47,54,55,56,58,63] and six multivariable studies [49,50,55,58,64,65]. Fourteen of the 33 studies concluded that a higher educational level of parents was a positive factor in accepting vaccination of COVID-19 for their children, with three studies [47,58,65] reporting the opposite outcome. Sex [39,47,48,50,57,63,64,65], age of the parents [39,44,49,50,54,55,65], household income [35,40,44,48,50,54,56,64,65] and parents’ occupation related to health-care [38,44,54,58,59] also appeared in both multivariable and univariable analysis as influencing factors in the COVID-19 vaccination decision. Participants who generally identified as male, older, higher-earning and worked as health-care workers were likely to show positive attitudes towards getting their children vaccinated. Participants with more children [40,44,54], USA republican party voters [42,53], Black, Asian and minority ethnic (BAME) [40,42,50,53], uninsured families [41,44], parents with abnormal mental states [53,57], and living with high-risk family members [55,58] were negative factors influencing parents’ decision to vaccinate their children. Additional factors, such as parents’ employment status [40], COVID-19 infection status [41], religious beliefs [43] and children’s chronic diseases [39,60], were identified as influencing factors in the vaccination decision in specific studies, which tested the factors using either multivariable or univariable analysis.

Subjective factors in Table 4 were mostly related to personal positions and attitudes toward vaccines and the epidemic, including willingness to vaccinate family members against flu/other diseases [38,39,41,44,50,54,58], willingness to vaccinate themselves against COVID-19 [53,54,59], fear of COVID-19 infection [39,44,47,50,54], fear of a new outbreak/persistence of the epidemic [38,58,59], trust in vaccines [54,59,60,62], the source of information related to vaccines [49,54,59], support for COVID-19 policies [49,55], and participants’ satisfaction with their society’s environment [44,53]. These factors have appeared in more than two articles and were similar in the conclusions from univariable and multivariable analysis. The reviewed articles showed that individuals who were willing to vaccinate family members against flu/other diseases, willing to vaccinate themselves against COVID-19, had a fear of getting COVID-19 infected and fear of a new outbreak/persistence of the epidemic, trusted vaccines, supported COVID-19 policies and were satisfied with their society’s environment were more likely to decide to COVID-19 vaccinate their children. In some studies, people who were exposed to information related to vaccines in the web/social media showed positive attitudes to child vaccination [54,59], while one study did not [49]. Some studies revealed study-specific factors. For example, Goldman et al. [39] argued that parents whose children were up-to-date on their vaccines were more willing to COVID-19 vaccinate their children; Yılmaz et al. [54] found that those who would recommend others to get vaccinated and those who believed that everyone should get vaccinated for herd immunity held more positive attitudes to vaccinating their children; and one study [55] argued that the characteristics of confidence in one’s knowledge about safety measures and regular information seeking about the pandemic were related to child vaccination willingness. Factors like the concerns about the side effects of vaccines [59,60], and parental willingness to enroll children in COVID-19 vaccine clinical trials [38,54] reported opposite results tested by multivariable analysis, but factors such as the types of vaccines [47] and the regions [63] were only tested by univariable statistical analysis.

### 3.4. Parents’ Intention to Vaccinate Children against COVID-19

**Figure 2:**
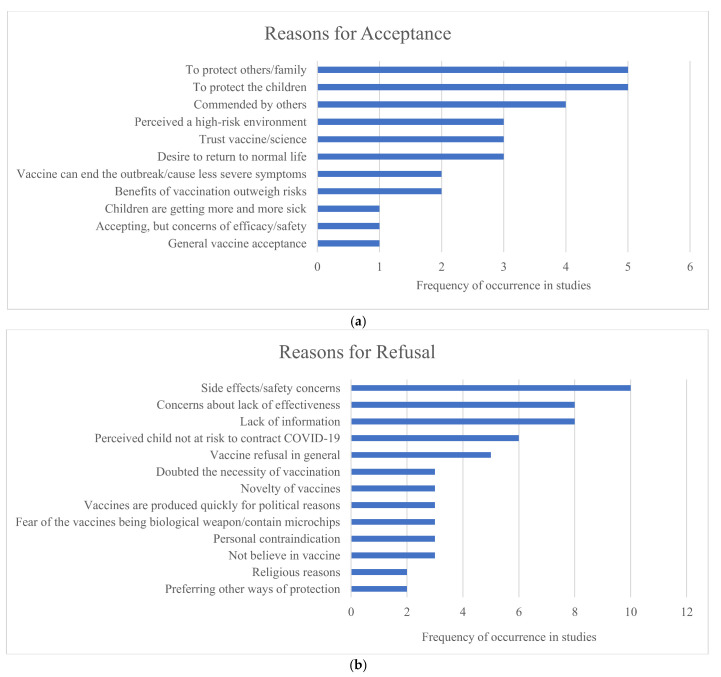
Frequency of reasons of parents’ intention to vaccinate children against COVID-19: (**a**) frequency of reasons for vaccine acceptance; and (**b**) frequency of reasons for vaccine refusal.

Among the 35 studies, 12 stated the reasons why parents were willing/unwilling to vaccinate their children against COVID-19. From the 12 studies, we summarized the reasons to vaccinate/not vaccinate and used the number of articles displaying a reason to calculate the frequency in Figure 2, where Figure 2a showed the frequency of reasons for acceptance and Figure 2b showed the frequency of reasons for rejection. From Figure 2a, to protect family/others/children was the most common reason for COVID-19 vaccination acceptance, which varied from 9.7% to 66.2% of respondents [39,40,41,53,54]. A significant number of respondents, ranging between 3% and 64.4%, were willing to vaccine their children when they perceived a high-risk environment and advice from others [39,40,49,54,63]. Trust in science and vaccines and the desire to return to a normal life were also identified in four articles [39,40,41,53], but the overall proportion of individuals who agreed to this view was only 14.6% at most. The advantages of vaccines, such as “vaccine can end the outbreak/cause less severe symptoms” and “benefits of vaccination outweigh risks” were widely reported [40,41,54], with one study finding 75.5% of the respondents giving this answer [54]. Other reasons reported for intention to vaccinate children were general vaccine acceptance [39] and increase of the number of children infected [54]. Some respondents indicated a willingness to vaccinate while still expressing concerns about the efficacy and safety of COVID-19 vaccines [39].

The reasons for resisting COVID-19 vaccines for children were focused on side effects and safety, as shown in Figure 2b [39,40,41,44,47,53,54,61,64,67], expressed by more than 20% of respondents in a majority of the studies. This was followed by concerns about the lack of vaccine effectiveness [40,41,44,47,54,61,64,67], with eight studies cited a lack of vaccine-related information leading to vaccine hesitancy [39,40,41,44,47,53,54,61]. While this was a common concern, never more than 40% of respondents reported the lack of COVID-19 vaccination information. Unexpectedly, no more than 31.2% of parents would refuse the vaccine when they perceived their children not at risk of contracting COVID-19 [39,40,47,53,54,64]. Not surprisingly, respondents refused the COVID-19 vaccines because of their general vaccine refusal [39,41,53,54,67], sometimes reaching 12.6% of respondents. In addition, concerns about novelty [39,40,41], personal contraindication [39,49,53], distrust of vaccines [47,54,67] and religious reasons [47,64] were also mentioned by respondents in their vaccination decision. We found that some factors occurred more frequently or only in certain countries, such as doubts about the necessity of vaccine [41,47,54], fear of the vaccines being biological weapon/containing microchips [41,47,54] and preferring other ways of protection [41,54] reported in the Turkey studies and worries that vaccines are produced too quickly for political reasons [44,53,61], most often mentioned in the USA studies.

## 4. Discussion

Our systematic review of the existing literature on parents’ decision-making on vaccinating their children identified the subjective and objective influencing factors in the vaccination decision to help health policy and government vaccination decision-making. We assessed 35 articles on parents’ attitudes to vaccinating children against COVID-19. Overall, the median unadjusted parents’ willingness rate to vaccinate children against COVID-19 was 59.3%, and the median willingness rate of parents to vaccinate themselves was 61.4%. Most of the literature showed that parents were more cautious about vaccinating their children [35,40,41,42,47,50,51,52,54,55,57,58,59,67,68,69,70] than vaccinating themselves. In addition, due to the different medical system backgrounds and composition of studies in different countries, there is great heterogeneity among the willingness rates making direct comparisons difficult. Differences among respondents across regions reflected different COVID-19 policies and cultural backgrounds [71]. For example, 92% of Zambia parents tended to vaccinate their children [62], a cross-sectional study from Turkey showed an 20–85 year vaccination rate of only 10.4% [68], and only 24.3% of Amish families wanted to get their children vaccinated [43]. Overall, we recommend that diverse interventions should be taken to improve parents’ willingness to vaccinate their children in different countries and areas considering the varied COVID-19 willingness rates and backgrounds. 

The objective factors influencing parental attitudes, respondents’ education level [41,42,44,47,49,50,54,55,56,58,63,64,65], sex [39,47,48,50,57,63,64,65], age [39,44,49,50,54,55,65], and race [40,42,50,53] remained the most reported factors for parents’ decisions to vaccinate their children against COVID-19 [71]. Respondents with lower education, who were female, of younger age or those who were in the BAME group were generally more cautious about COVID-19 vaccines for children than other groups. It could be explained by the fact that higher education, such as Master’ s degree and post-graduate degrees, was associated with decreased vaccine risk perceptions [65] and better informed groups tended to be more caring about their health and well-being [72]. Parental experience was important, with women more hesitant than men in vaccinating generally [73]. As the primary caregiver of children in families, mothers should be the focus of COVID-19 vaccine promotion and BAME groups should also be a special vaccine education target group. By targeting these groups, public health campaigns can raise vaccination awareness to protect children and families. Since the fatality rate of young people was lower than that of the elderly [74], young people may have a lower risk perception of the epidemic, which highlights the importance for young parents to correctly understand the key role of themselves in the spread of the epidemic and family protection. The interpretation of race should be deliberative because the literature on this subject is sparse, although there is evidence that BAME groups and people living in the most deprived areas are at high risk of acquiring COVID-19 infection and at increased risk of death from COVID-19 [40]. It is important not to racially profile BAME groups, since emphasizing vaccine hesitancy risks taking a victim-blaming perspective on race [75]. Governments and medical institutions should make vaccines easily accessible to BAME groups, perhaps with local distribution points connected to religious, sporting and other community centers. One policy recommendation is to leverage trusted community leaders to engage communities of color in public health campaigns [76].

Parents with more children [40,44,54], who were unemployed [40] and those with lower income [35,40,44,48,50,54,56,64,65] and no insurance [41,44] have been reported to be more likely to refuse the vaccine, which might reflect financial distress. One constraint is vaccine accessibility, with only 3.1% of people in low-income countries having received at least one dose [77]. Akarsu [41] also found that the proportion of people willing to vaccinate their children increases when the vaccine was free. Therefore, to expand the vaccine coverage, policies need to address vaccine accessibility for the poor. Some studies suggest that parents tended to refuse vaccinations for children with chronic diseases [39,60] and those in younger age groups [39,49], that possibly reflects safety concerns. More research and information are required to address the issue of vaccine side effects in these groups [78]; before that, these children could strengthen physical protection measures and be protected in a safe environment by vaccinating people around them.

We found that the subjective factors influencing parental attitudes towards COVID-19 vaccines for children were mainly related to personal positions and attitudes towards vaccines and the epidemic of COVID-19. Parents who believed in vaccines [54,59,60,62], supported vaccination policies [49,55], and felt more satisfied with their society [44,53], showed a higher tendency to get their children vaccinated. These factors are related to people’s attitude towards politics and science, which indicates the importance of people’s trust and support for policies and society. A trusted government and social environment can play a positive role in the development of herd immunity [79,80]. One study [54] found that people who were willing to get their children vaccinated, also preferred to recommend others to get vaccinated. The numbers of parents who wished to see all members of society vaccinated for herd immunity were significantly higher among those who were willing to allow the COVID-19 vaccine to be given to their children. Therefore, parents with such characteristics could be marshalled to become community health service volunteers, provided with professional vaccine knowledge training, and engaged to play an important role in community vaccine publicity and education through active communication with residents.

People who feared to get infected by coronavirus [39,44,47,50,54], feared a new COVID-19 outbreak or were concerned about the persistence of the epidemic [38,58,59], showed a positive correlation towards vaccinating. This means that people who are more alert and concerned about the epidemic were more inclined to seek the protection of vaccine, which suggests increasing the vigilance of the population by releasing information about the development of the epidemic through official institutions might increase vaccine acceptance. Notably, some parents may refuse to vaccinate their children because they perceive that their children are not at risk for COVID-19 [39,40,47,53,54,64], and this idea might be one of the reasons for recent rising infection rates among children [2]. To raise parents’ awareness, campaigns should provide more information about risks and hazards associated with children infected with COVID-19.

The role of social media is unclear. In some studies [54,59], those who obtained COVID-19 information through social media showed an active intention to vaccinate their children, while other studies [49] indicated social media active people were unwilling to have their children vaccinated, which highlights the complexity of the information dissemination. When people receive information about the benefits of vaccines, they will be more inclined to accept the vaccine, and if they receive more negative information about the vaccine, they would be less likely to get vaccinated, and this indirectly explains the contradictory role of the social media played. Unfortunately, we found a sentiment analysis [81] that concluded negative tweets populate pro- and anti-vaccine communities, thus confirming the popularity of negative sentiment on social media. At present, one of the greatest risks to human health comes from the deluge of misleading, conflicting, and manipulated information currently available online, including health misinformation. Vaccination is a topic particularly susceptible to online misinformation [82]. Information about vaccines on social media platforms should be more strictly supervised and managed to avoid the wide spread of false information and ensure that the public can receive authentic and effective COVID-19 related information [83]. The control of social media, and the exclusion of misleading information, raises issues of free access to information and freedom of the internet. The types of controls on vaccine and COVID-19 (mis)information will depend on the country-specific rules on information and social media access, making overall recommendations on social media difficult. The challenge is to use social media to provide accurate information on the benefits of vaccination, especially for children. 

Some respondents were afraid to have their children vaccinated because of the side effects of the vaccine [59,60], which was also the top reason for rejecting the vaccine [39,40,41,44,47,53,54,61,64,67]. In addition to improving the quality of the vaccine itself, authorities should strengthen surveillance and management of COVID-19 vaccines and make the process transparent, and conduct further research on vaccine contraindications and adverse reactions. Increasing public confidence in COVID-19 vaccines and the need for vaccines may be an effective way to improve vaccination coverage. To promote the COVID-19 vaccine, concerted effort by healthcare workers is required [84]. An education campaign may be necessary for healthcare workers to improve their knowledge about the epidemic and COVID-19 vaccines, improve their ability to explain the effects of vaccines patiently and correctly, and enhance their responsibilities for monitoring the vaccination process. Healthcare workers are on the front line, so it is crucial that they are able to provide community health education on COVID-19 vaccination issues and interview parents with vaccine hesitation to increase child vaccination rates [85]. 

There are some parental vaccination decision-making reasons that occurred only in specific countries, such as doubt about the necessity of vaccines [41,47,54], fear that vaccines were biological weapons/contained microchips [41,47,54], and preferred other means to protect children [41,54]. For example, respondents from the USA. frequently mentioned that they were concerned about the COVID-19 vaccines being produced rapidly for political reasons [44,53,61]. These studies point to the need to tailor policy responses to the socio-political environment of each country. One recommendation is that countries with developed vaccination infrastructure for the child and adolescent could consider integrating COVID-19 vaccines into the existing routine immunization programs.

### Strengths and Limitations

This paper is the first assessment of the extant global literature on parents’ decisions to COVID-19 vaccinate their children. Our analysis reported the univariate and multivariate statistical results used in different studies by dividing the influencing factors into objective and subjective factors. Our review has some limitations. First, most of the included studies were cross-sectional studies, so the association between influencing factors and vaccination intention cannot be explained from the perspective of causality. Second, most of these studies were nonprobability-based sampling, which may lead to selection bias. Third, parents may have recall bias when filling in the questionnaire themselves, which may also affect the accuracy of the papers’ results. Fourth, methodologically, due to the limitations of the volume and heterogeneity of published literature, there is no strict meta-analysis in our study.

## 5. Conclusions

We found that the median rate of parents willing to vaccinate their children was 59.3%. While vaccination intention rates were highly heterogeneous across countries, the factors influencing parents’ attitudes towards children’s vaccination were similar. Parents’ education level was the most important factor, but sex, age, and household income were also key factors in the vaccination decision. Among all the reasons for vaccinating, or not, protecting children, family and others was the leading reason to vaccinate, and the fear of side effects and safety were the top reasons to not vaccinate. The most important policy recommendations are for healthcare workers and government to create an informed and transparent environment for the rollout of COVID-19 vaccines, to ensure the accuracy and timeliness of COVID-19-related information, and to carry out targeted publicity and education campaigns. These key policy directions can reinforce vaccine adherence and address vaccine hesitancy.

## Figures and Tables

**Table 1:** Search strategy algorithms.

Search	Keywords
#1	COVID-19 OR SARS-CoV-2 OR novel coronavirus OR coronavirus disease
#2	vaccin* OR immunization
#3	child* OR mother OR parents OR kid
#4	Acceptance OR Agreement OR Willingness OR Refusal OR Resistance OR Confidence OR Hesitancy OR Antivaxx OR Antivaxxers OR Antivaccine OR Anti-vaccine
#5	2019 OR 2020 OR 2021
#6	#1 AND #2 AND #3 AND #4 AND #5

**Table 2:** Characteristic of Studies.

First Author	Date of Publication	Sample Size	Participants	Parents’ Age	Parents’ Sex (Female)	Country	The Rate to Vaccinate Children against COVID-19	The Rate to Vaccinate Parents Themselves against COVID-19
Amiel A. Dror [35]	12 August 2020	1941	Healthcare workers and general population	NA	NA	Israeli	70% for general population 60% for doctors 55% for nurses	75% for general population 78% for doctors 61% for nurses
Pınar Yılmazbaş [37]	29 September 2020	440	Parents	39.1 ± 6.4	70.4%	Turkey	73.90%	NA
Luca Pierantoni [38]	12 October 2020	1812 families	Parents	NA	NA	Italy	Recommended (91.1%)	NA
Ran D. Goldman [39]	10 November 2020	1541	Caregivers of child patients	39.9 (median) (SD 7.58)	71.97%	USA, Canada, Israel, Japan, Spain, and Switzerland	65.20%	NA
Sadie Bell [40]	17 November 2020	1252	Parents and guardians	32.95 ± 4.565	95%	England	89.10%	90.10%
Büşra Akarsu [41]	5 December 2020	759 (232 had children between the ages of 0–18)	Adults	32.41 ± 9.92	62.8%	Turkey	38.4% 41.9% (If free)	49.7% 55.5% (If free)
Emily A. Largent [42]	18 December 2020	2724	Adults	>18	45.9%	USA	48.60%	61.40%
Ethan M. Scott [43]	12 February 2021	391	Amish families	38 (median)	67%	USA	24.30%	NA
Malia Skjefte [44]	1 March 2021	17871(5294 pregnant women)	Pregnant women and mothers	34.4 ± 7.3	100%	Global	Given a 90% COVID-19 vaccine efficacy:69.2%	Given a 90% COVID-19 vaccine efficacy: 52.0% (pregnant women) 73.4% (non-pregnant women)
Jorge L. Alvarado-Socarras [45]	19 March 2021	1066	Physicians	Inconsistent between groups	47%	Colombia	85.70%	84.60%
Ronnie R. Marquez [46]	24 March 2021	99	Caregivers of children receiving oral healthcare	38.8 ± 9.1	83.5%	USA	21.60%	19.60%
Yigit, Metin [47]	1 April 2021	428	The parents had children who were inpatients or outpatient	39.7 ± 10.7	63.5%	Turkey	28.9% (foreign vaccine) 56.8% (national vaccine)	33.9% (foreign vaccine) 62.6% (national vaccine)
Qiang Wang and Shixin Xiu [48]	1 April 2021	3009	Parents and HCWs from immunization clinics	31.36 ± 4.46	74.6%	China	59.30%	51.20%
Marco Montalti [49]	10 April 2021	4993	Parents/guardians	40–49 majority (55.4%)	76.6%	Italy	60.40%	NA
Bridget J. Kelly [50]	12 April 2021	2279 (27% of respondents had children)	Adults	50–64 majority (26%)	52%	USA	52.70%	80.5% (male) 73.9% (female)
Erdem Gönüllü [51]	16 April 2021	506 (379 having a child)	Pediatrics	41 ± 8	58%	Turkey	75%	83%
Jia Lu [52]	7 May 2021	3673	Parents of the students	NA	69.1%	China	31.3~87.5%	33.5%~89.7%
Stephanie Milan [53]	10 May 2021	240	Mothers with a mental health history	36.9 ± 7.42	100%	USA	38.7% of mothers with a PTSD history were reluctant versus 25.8% of mothers without a PTSD history	Among mothers with a PTSD history, 40% were vaccine reluctant for themselves versus 23.9% of mothers without a PTSD history
Meltem Yılmazp [54]	16 May 2021	1035	Parents	30–39 years old (53.3%)	77.8%	Turkey	36.30%	59.90%
Susanne Brandstetter [55]	17 May 2021	612 families	Parents	NA	80% by mothers, and 10% by mothers and fathers together	Germany	51%	58%
Erin Hetherington [56]	21 May 2021	1321	Parents	42.2 ± 4.4	100%	Canada	60.40%	NA
Linda Thunström [70]	4 June 2021	3133	Adults	45.63 ± 16.52	51.9%	USA	19.7% (not intend to vaccinate)	19.5% (not intend to vaccinate)
Yucheng Xu [57]	6 June 2021	4748	Parents	40.28 ± 5.08	76.0%	China	72.70%	74.80%
Yehong Zhou [58]	9 June 2021	1071 (at least have 747 children)	Adults and guardians of children who visited community health centers	34.0 ± 7.4	76.5%	China	85.30%	88.60%
Zixin Wang [59]	17 June 2021	1332	Parents who are healthcare workers	31–40 majority (61.30%)	89.4%	China	44.50%	72.40%
Kristine M. Ruggiero [60]	30 June 2021	427	Parents of school-age children	NA	NA	NA	49.45%	44.17%
Kimberly K. Walker [61]	30 June 2021	25	Mothers	40–49 majority (60.00%)	100%	USA	16%	16%
Andrea C. Carcelen [62]	6 July 2021	2400	Parents who brought their children to vaccinate MR vaccine	NA	NA	Zambia	92%	66%
Aaron M Scherer [63]	16 July 2021	1022	Parents and guardians	NA	48.2%	USA	55.50%	NA
Chloe A. Teasdale PhDab [64]	17 July 2021	2074	Primary caregivers	30–44 majority (66.88%)	61.23%	USA	50.30%	49.40%
Kaidi He [65]	23 July 2021	252	Parents of children patients	30–44 majority (55.2%)	83.3%	USA	NA	NA
Matthew Greenhawt [66]	24 February 2021	4855	Adults	30–39 majority (17.2%)	50.2%	USA	70.10%	65.70%
Reem Al-Mulla [67]	18 June 2021	462	QU students aged 18 years and above	18–24 majority (32.7%)	62.6%	QATAR	46% (not intend to vaccinate)	62.6%
Hatice İkiışık [68]	11 May 2021	384	Adults ages of 20 to 85	43.3 ± 13.5	47.4%	Turkey	10.40%	54.70%
Flora Fedele [69]	7 June 2021	640	Parents attending 4 pediatric practices	35–50 majority (59.4%)	74%	Italy	17.20%	26.50%

**Table 3:** Objective factors influencing parents’ decision to vaccinate children against COVID-19.

Authors	Characteristic in Univariable Analysis	Characteristic in Multivariable Analysis	Positive/Negative
Luca Pierantoni [38]		Either parent is a health-care worker	P
Ran D. Goldman [39]	Older children; When fathers completed the survey; If the caregiver was older	Older children	P
		Child has chronic illness Mother completing the survey	N
Sadie Bell [40]	Homemaker/unemployed (ref: working full-time); Lower Income < GBP 35,000 (ref: medium income GBP 35,000–84,999); More than 4 children (ref: 1 child); Black, Asian, Chinese, Mixed or other ethnicity (ref: white)	Low Income < GBP 35,000 (ref: medium income GBP 35,000–84,999); More than 4 children (ref: 1 child); Black, Asian, Chinese, Mixed or other ethnicity (ref: White)	N
Büşra Akarsu [41]	The increasing level of education; Who have SSI or private health insurance; Infection Status with COVID-19		P
Emily A. Largent [42]	Democrats (ref: Republicans and Independents); Respondents with a bachelor’s degree or higher (ref: less than a bachelor’s degree)		P
	Black respondents (ref: Non-Black respondents)		N
Ethan M. Scott [43]	Swartzentruber Amish		N
Malia Skjefte [44]	Master’s, professional school, doctoral degree (ref: college diploma or equivalent); Middle class to wealthy (ref: lower middle class to poor) Physicians (ref: non-essential workers); Have health insurance (ref: no health insurance)	Have health insurance (ref: no health insurance)	P
	Lower than 40 (ref: 40–65 years); Two or more children (ref: no child)	Lower than 40 (ref: 40–65 years); Middle class to wealthy (ref: lower middle class to poor)	N
Yigit, Metin [47]	Parents whose fear and anxiety levels were high		P
	As the education level increased, parents were less likely to		N
Qiang Wang & Shixin Xiu [48]	College education or below (ref: Master’s Diploma or above); Parents having annual household income RMB 50,000–<150,000 (ref: RMB >= 150,000)	College education or below (ref: Master’s Diploma or above)	P
Marco Montalti [49]		Children aged 6–10 years (ref: >= 14); Parents <= 29 years old (ref: >= 50); Parents with low educational level	N
Bridget J. Kelly [50]		Hispanic origin	P
		Female; People with young age between 25–64 (ref: 65+); High school or less (ref: bachelor’s degree or higher); Black (ref: White); Income < USD 50,000 (ref: >=USD 150,000)	N
Stephanie Milan [53]	Maternal education;	Maternal education;	P
	African-American; Republican; PTSD/Lifetime PTEs	African-American; Republican	N
Meltem Yılmaz [54]	Parents aged 40 or older (ref: 18–29); Educated to university level or higher (ref: high school or lower); With high economic status; Parents being healthcare workers; With only one child (ref: three or more)	Parents being healthcare workers	P
Susanne Brandstetter [55]	Higher mother’ s age; High educational level (university entrance level) (ref: Medium educational (10 years of schooling))	High educational level (university entrance level) (ref: Medium educational (10 years of schooling))	P
	High educational level (university entrance level) (ref: Medium educational (10 years of schooling))		
	Risk group member in family, friends (yes)	Risk group member in family, friends (yes)	N
Erin Hetherington [56]	Participants with lower education, lower income		N
Yucheng Xu [57]	Male parents		P
	Parents with psychological distress	Parents with psychological distress	N
Yehong Zhou [58]	Participants with older individuals in their families; Participants with Bachelor’s degrees or higher; Participants with healthcare-related occupations	Participants with older individuals in their families; Participants with higher levels of education; Participants with healthcare-related occupations	N
Zixin Wang [59]	Worked in the infectious disease departments		P
	Those had middle rank technical job title		N
Kristine M. Ruggiero [60]		High-risk child (chronic condition)	P
Aaron M Scherer [63]	Female; Hispanic; Who had less than a bachelor’s degree; Living in the Midwest or South Census regions		N
Chloe A. Teasdale [64]		Asian parents (Ref: Non-Hispanic white)	P
		Female (Ref: male); Had lower educational attainment (high school education or less); Had household income USD 25,000–49,000 (ref: >= USD 100,000)	N
Kaidi He [65]		Male sex; Age 45–54 years (Ref: 18–29 years); Less than High School education; Household income > 100 K (Ref: <49 K)	P

**Table 4:** Subjective factors influencing parents’ decision to vaccinate children against COVID-19.

Authors	Characteristic in Univariable Analysis	Characteristic in Multivariable Analysis	Positive/Negative
Luca Pierantoni [38]		Fear of a new outbreak moderately (ref: Not at all/A little); Will get child vaccinated against flu; Will enroll child in a COVID-19 vaccine clinical trial	P
Ran D. Goldman [39]	Children that were up-to-date on their vaccines; If the child or the caregiver reported they were immunized against influenza in the last year; If the caregiver was more concerned about their child or themselves having COVID-19 when arriving to the ED	Children that were up-to-date on their vaccines; If the child or the caregiver reported they were immunized against influenza in the last year; Caregiver concern that the child had COVID-19	P
Büşra Akarsu [41]	Who got seasonal flu vaccine	Perceived risk of the virus/precautions	P
Malia Skjefte [44]	Negative experiences with COVID-19; Past acceptance and perceived safety/efficacy of other vaccines; Confidence in COVID-19 vaccine; Perceived risk of the virus/precautions; Public trust and satisfaction	Past acceptance and perceived safety/efficacy of other vaccines; Confidence in COVID-19 vaccine; Public trust and satisfaction	P
Yigit, Metin [47]	Preference for the foreign vaccine for children was higher in males; Preference for the domestic vaccine (ref: foreign vaccine)		P
	Accept the domestic vaccine for their children		N
Marco Montalti [49]		Relying on information found in the web/social media; Disliking mandatory vaccination policies	N
Bridget J. Kelly [50]		Received flu vaccine in past year; Worried about getting the coronavirus; High/very high perceived threat from the coronavirus	P
Stephanie Milan [53]	Benevolent view of world; Mother and child vaccine intentions were highly correlated		P
	Institutional distrust	Institutional distrust	N
Meltem Yılmaz [54]	parents’ willingness and positive attitudes towards the COVID-19 vaccine; In the event of a post-mutation increase in COVID-19 related mortality in children; Whose children had received paid-for vaccines in addition to the Expanded Program on Immunisation; Who said they would advise others to receive the COVID-19 vaccine; Who were worried about themselves or their children contracting COVID-19; Who agreed that the COVID-19 vaccine would end the pandemic; Who agreed that everyone should be vaccinated against COVID-19 for herd immunity; Who were exposed to information related to the COVID-19 vaccine in the social media in the previous month	Parents’ willingness to receive the vaccine and positive attitudes toward it; Willing to participate in the COVID-19 vaccine trial; Willing to allow their children to participate in a COVID-19 vaccine trial; Willing to allow the COVID-19 vaccine to be given to their children if children catch COVID-19 and mortality increases following a mutation; Advising others to receive the COVID-19 vaccine; Worrying that they or their children may have COVID-19; Believing that the COVID-19 vaccine will end the pandemic; Stating that everyone should be vaccinated for herd immunity against COVID-19	P
Susanne Brandstetter [55]	Confidence in one’s knowledge about safety measures (0–6); Trust in policy measures (0–4); Regular information seeking about Corona pandemic (0–4)	Confidence in one’s knowledge about safety measures (0–6); Regular information seeking about Corona pandemic (0–4);	P
	Perception that policy measures are exaggerated (0–4)	Perception that policy measures are exaggerated (0–4)	N
Yehong Zhou [58]	Participants with a self-reported history of influenza vaccination; Prospect of COVID-19 persistence: Persistent (ref: Transient or short-term presence)	Participants with a self-reported history of influenza vaccination	P
Zixin Wang [59]		Perceived higher vaccine efficacy and longer protection duration; Perceived high/very high chance for China to prevent another wave of COVID-19 outbreak with COVID-19 vaccines in place; Willingness to receive a COVID-19 vaccination for themselves; Higher frequency of information exposure through social media and interpersonal communication related to COVID-19 vaccination	P
		Knowing some people who experienced serious side effects following COVID-19 vaccination	N
Kristine M. Ruggiero [60]		Trust information about shots;	P
		Overall hesitancy about childhood shots; Better for children to get few vaccines at the same time; Concerned for serious side effect; Get shots so child can enter daycare or school; Concerned COVID vaccine might not prevent disease	N
Andrea C. Carcelen [62]		Who believed COVID-19 vaccines would be safe; Who believed COVID-19 vaccines would be effective	P

## Data Availability

The data presented in this study are available on request from the corresponding author.

## Appendix A

**Table A1:** Preferred Reporting Items for Systematic Reviews and Meta-Analyses extension for Scoping Reviews. (PRISMA-ScR) Checklist.

SECTION	ITEM	PRISMA-ScR CHECKLIST ITEM	REPORTED ON PAGE #
TITLE			
Title	1	Identify the report as a scoping review.	1
ABSTRACT			
Structured summary	2	Provide a structured summary that includes (as applicable): background, objectives, eligibility criteria, sources of evidence, charting methods, results, and conclusions that relate to the review questions and objectives.	1
INTRODUCTION			
Rationale	3	Describe the rationale for the review in the context of what is already known. Explain why the review questions/objectives lend themselves to a scoping review approach.	2
Objectives	4	Provide an explicit statement of the questions and objectives being addressed with reference to their key elements (e.g., population or participants, concepts, and context) or other relevant key elements used to conceptualize the review questions and/or objectives.	2
METHODS			
Protocol and registration	5	Indicate whether a review protocol exists; state if and where it can be accessed (e.g., a Web address); and if available, provide registration information, including the registration number.	2
Eligibility criteria	6	Specify characteristics of the sources of evidence used as eligibility criteria (e.g., years considered, language, and publication status), and provide a rationale.	3
Information sources *	7	Describe all information sources in the search (e.g., databases with dates of coverage and contact with authors to identify additional sources), as well as the date the most recent search was executed.	3
Search	8	Present the full electronic search strategy for at least 1 database, including any limits used, such that it could be repeated.	3
Selection of sources of evidence †	9	State the process for selecting sources of evidence (i.e., screening and eligibility) included in the scoping review.	3
Data charting process ‡	10	Describe the methods of charting data from the included sources of evidence (e.g., calibrated forms or forms that have been tested by the team before their use, and whether data charting was done independently or in duplicate) and any processes for obtaining and confirming data from investigators.	3
Data items	11	List and define all variables for which data were sought and any assumptions and simplifications made.	3
Critical appraisal of individual sources of evidence §	12	If done, provide a rationale for conducting a critical appraisal of included sources of evidence; describe the methods used and how this information was used in any data synthesis (if appropriate).	NA
Synthesis of results	13	Describe the methods of handling and summarizing the data that were charted.	3
RESULTS			
Selection of sources of evidence	14	Give numbers of sources of evidence screened, assessed for eligibility, and included in the review, with reasons for exclusions at each stage, ideally using a flow diagram.	4
Characteristics of sources of evidence	15	For each source of evidence, present characteristics for which data were charted and provide the citations.	5
Critical appraisal within sources of evidence	16	If done, present data on critical appraisal of included sources of evidence (see item 12).	NA
Results of individual sources of evidence	17	For each included source of evidence, present the relevant data that were charted that relate to the review questions and objectives.	7
Synthesis of results	18	Summarize and/or present the charting results as they relate to the review questions and objectives.	12
DISCUSSION			
Summary of evidence	19	Summarize the main results (including an overview of concepts, themes, and types of evidence available), link to the review questions and objectives, and consider the relevance to key groups.	23
Limitations	20	Discuss the limitations of the scoping review process.	26
Conclusions	21	Provide a general interpretation of the results with respect to the review questions and objectives, as well as potential implications and/or next steps.	26
FUNDING			
Funding	22	Describe sources of funding for the included sources of evidence, as well as sources of funding for the scoping review. Describe the role of the funders of the scoping review.	27

JBI = Joanna Briggs Institute; PRISMA-ScR = Preferred Reporting Items for Systematic reviews and Meta-Analyses extension for Scoping Reviews. * Where sources of evidence (see second footnote) are compiled from, such as bibliographic databases, social media platforms, and Web sites. † A more inclusive/heterogeneous term used to account for the different types of evidence or data sources (e.g., quantitative and/or qualitative research, expert opinion, and policy documents) that may be eligible in a scoping review as opposed to only studies. This is not to be confused with *information sources* (see first footnote). ‡ The frameworks by Arksey and O’Malley (6), Levac and colleagues (7), and the JBI guidance (4, 5) refer to the process of data extraction in a scoping review as data charting. § The process of systematically examining research evidence to assess its validity, results, and relevance before using it to inform a decision. This term is used for items 12 and 19 instead of “risk of bias” (which is more applicable to systematic reviews of interventions) to include and acknowledge the various sources of evidence that may be used in a scoping review (e.g., quantitative and/or qualitative research, expert opinion, and policy document).
